# Whole-lung finite-element models for mechanical ventilation and respiratory research applications

**DOI:** 10.3389/fphys.2022.984286

**Published:** 2022-10-04

**Authors:** Nibaldo Avilés-Rojas, Daniel E. Hurtado

**Affiliations:** ^1^ Department of Structural and Geotechnical Engineering, School of Engineering, Pontificia Universidad Católica de Chile, Santiago, Chile; ^2^ Institute for Biological and Medical Engineering, Schools of Engineering, Medicine and Biological Sciences, Pontificia Universidad Católica de Chile, Santiago, Chile

**Keywords:** mechanical ventilation, lung modeling, respiratory mechanics, poroelasticity, pressure-volume curve

## Abstract

Mechanical ventilation has been a vital treatment for Covid-19 patients with respiratory failure. Lungs assisted with mechanical ventilators present a wide variability in their response that strongly depends on air-tissue interactions, which motivates the creation of simulation tools to enhance the design of ventilatory protocols. In this work, we aim to create anatomical computational models of the lungs that predict clinically-relevant respiratory variables. To this end, we formulate a continuum poromechanical framework that seamlessly accounts for the air-tissue interaction in the lung parenchyma. Based on this formulation, we construct anatomical finite-element models of the human lungs from computed-tomography images. We simulate the 3D response of lungs connected to mechanical ventilation, from which we recover physiological parameters of high clinical relevance. In particular, we provide a framework to estimate respiratory-system compliance and resistance from continuum lung dynamic simulations. We further study our computational framework in the simulation of the supersyringe method to construct pressure-volume curves. In addition, we run these simulations using several state-of-the-art lung tissue models to understand how the choice of constitutive models impacts the whole-organ mechanical response. We show that the proposed lung model predicts physiological variables, such as airway pressure, flow and volume, that capture many distinctive features observed in mechanical ventilation and the supersyringe method. We further conclude that some constitutive lung tissue models may not adequately capture the physiological behavior of lungs, as measured in terms of lung respiratory-system compliance. Our findings constitute a proof of concept that finite-element poromechanical models of the lungs can be predictive of clinically-relevant variables in respiratory medicine.

## 1 Introduction

With more than 550 million cases and 6 million deaths to date, the Covid-19 pandemic continues to be one of the most urgent health problems in the world (Worldometers, 2022). The most concerning complication of Covid-19 is acute respiratory failure, whose treatment demands invasive mechanical ventilation (MV) in up to 89.9% of patients admitted to intensive care units (Wunsch, 2020). Despite being the standard of care for many decades, there is still no consensus about optimal settings during MV treatment, as the respiratory response of patients presents high variability that can compromise the clinical outcome (Chiew et al., 2018; Morton et al., 2020; Grasselli et al., 2021). From this perspective, predictive patient-informed simulations of respiratory mechanics during MV and other pulmonary conditions arise as a unique opportunity to personalize care in critical and respiratory medicine, as they provide a safe framework to design, prototype, and test individualized ventilation protocols and interventions *in silico* (Chase et al., 2018; Zhou et al., 2021), with the aim of optimizing treatment and improving clinical outcomes.

Modeling the mechanics of the respiratory system has been traditionally approached from an engineering systems perspective (Bates, 2009). Compartment models have been proposed in the literature, where the respiratory system is represented as an interconnected network of elastic deformable elements (lungs/lobes) and resistive conduits (airways) (Maury, 2013; Arunachalam et al., 2020). This approach has the advantage of directly considering clinically-relevant variables such as airway pressure, volume, and flow, all of which can be tracked in real-time on patients undergoing mechanical ventilation with current monitoring technologies (Major et al., 2018; Morton et al., 2018). Single-compartment lung models are widely employed in the analysis of respiratory mechanics waveforms, as they are fitted to these physiological signals to estimate key lung parameters such as respiratory-system compliance, airway resistance, and expiratory time constants. These parameters are very relevant in clinical practice, supporting medical diagnosis in respiratory medicine and guiding clinical decisions in intensive care units (Hess, 2014).

Understanding regional ventilation mechanisms in the lung has motivated the extension of compartment models to represent the complex geometry of the airways. Fractal lumped models have been created using a flow dynamics simulations on reconstructions of the airway tree that connect at the terminals with acini (Tawhai and Bates, 2011; Swan et al., 2012; Roth et al., 2017a; Roth et al., 2017b; Pozin et al., 2017). While these models have been successful in the estimation of 3D distributions of ventilation and alveolar pressure, they fail, by construction, to seamlessly couple the interaction between deformation of alveolar tissue and air flow in the lung. This mechanical coupling is needed to establish a direct and accurate relation between regional lung ventilation and deformation, which motivates the creation of continuum approximations that consider both the solid and gas phases in the lung.

The tight interaction between the alveolar (porous) structure and the air pressure acting on the alveolar wall highlights the inherent poromechanical nature of lung function (Sarabia-Vallejos et al., 2021). The first attempts to model the transitional and respiratory regions of the lung using poroelastic continuum theory on idealized geometries was carried out by Kowalczyk (1993). Berger et al. (2016) extended this poroelastic formulation to create a 3D computational simulations of a lung under idealized spontaneous breathing. Using a Neo-Hookean material to represent the contribution of alveolar tissue, they were able to model the local coupling between alveolar pressure and tissue deformation in the lung. More recently, anatomical 3D finite-element simulations of the lungs have been developed to study their quasi-static poromechanical response (Patte et al., 2022b). The impact of material parameters on the pleural pressure arising from the interaction of the lungs with the thoracic cage was studied under static conditions. These contributions constitute a proof of concept that 3D poromechanical models of human lungs can be constructed, and that they deliver mechanically-consistent results. However, their applicability to clinically-relevant conditions, such as lungs under mechanical ventilation, remains unexplored.

Predictive mechanical simulations of whole organs necessitate accurate constitutive models of the underlying tissue. The non-linear mechanical response of the lung parenchyma has been described using several phenomenological hyperelastic constitutive laws, which include exponential strain energy densities (Fung, 1974; Tawhai et al., 2009; Ma et al., 2013), polynomials models (Berger et al., 2016; Yoshihara et al., 2017), and linear combinations of the former (Rausch et al., 2011; Birzle et al., 2018; Birzle et al., 2019). These phenomenological approximations require the determination of material constants for experimental data, which has been approached from uniaxial tensile experiments (Rausch et al., 2011; Bel-Brunon et al., 2014), biaxial stretching (Gao et al., 2006) and volumetric expansion tests (Birzle et al., 2018; Birzle et al., 2019). Despite the wide range of constitutive laws and experimental data available for lung tissue mechanics, only simple material models have been included in whole-lung simulations (Tawhai et al., 2009; Berger et al., 2016).

In this work, we formulate a continuum seamlessly coupled framework to construct anatomical computational models of the lungs, and explore its potential for clinically-relevant applications. Our guiding question is: Can we construct high-fidelity poromechanical lung models that capture the distinctive features of respiratory mechanics of lungs under mechanical ventilation? In addition, a second question that arises is: What is the impact of the choice of constitutive model on the large-scale organ response? To answer these questions, in Section 2 we develop a continuum finite-deformation poroelastic framework suitable for the construction of dynamic finite-element models of patient-specific geometries of the lungs and review two clinical applications in respiratory medicine, namely mechanical ventilation and the supersyringe method for quasi-static lung mechanics characterization. We further discuss the necessary modeling considerations adopted in this work to resemble these procedures. The numerical simulations of these two applications, along with the study of simulations under different constitutive models are reported in Section 3. We end by discussing the ability of our simulations to capture the behavior of mechanically-ventilated lungs, their comparison with clinical parameters reported in the literature, and the impact of the choice of constitutive models on our simulations in Section 4.

## 2 Materials and methods

### 2.1 Dynamic continuum poroelastic formulation of lung respiratory mechanics

**FIGURE 1:**
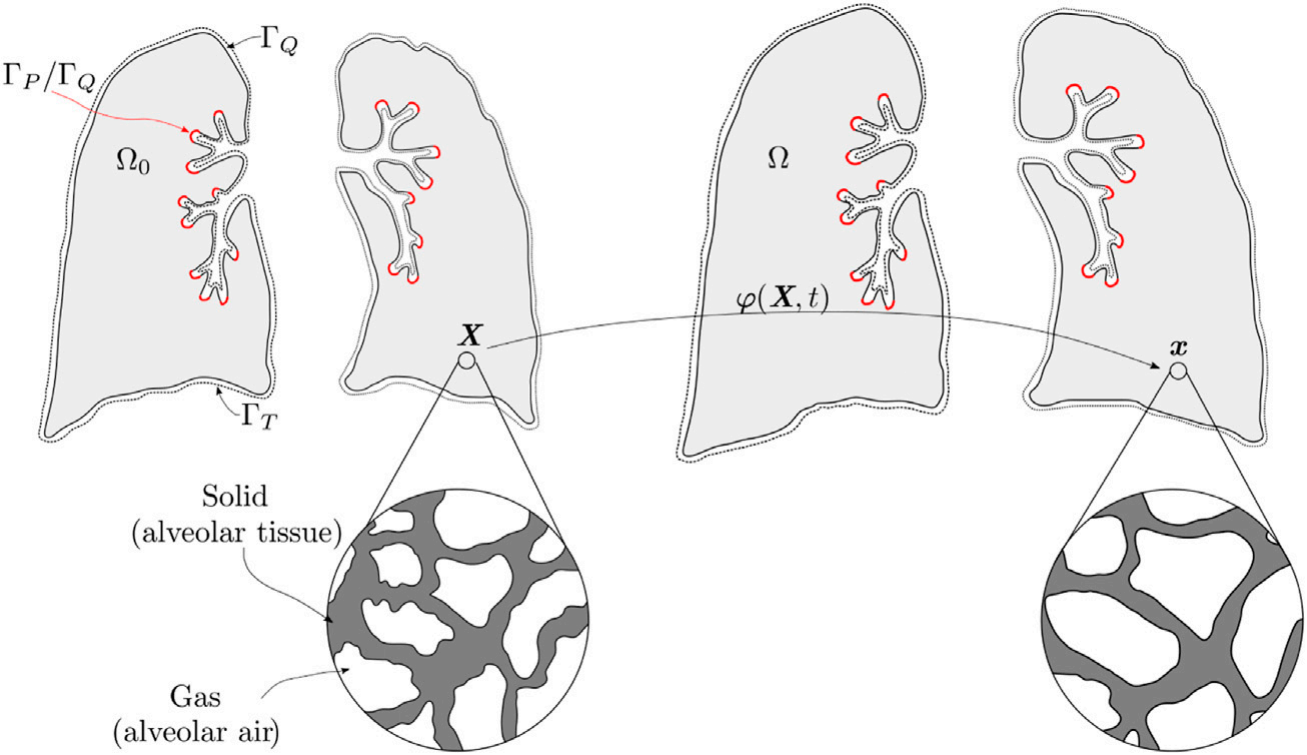
Material and spatial configuration of the lung continuum with porous composite idealization. The red color indicates the airway boundary, where it prescribes pressure or flow depending on the application; the complement of the surface is a null prescribed flow boundary. In addition, traction is prescribed on the whole surface.

In the following, we adopt a continuum approach to porous materials as described in Coussy (2004). To this end, the lung is represented by a continuum, whose microstructure is composed by a gas and a solid phase, see Figure 1. We will refer to the gas phase as the alveolar air, and to the solid as the alveolar tissue. Let Ω_0_ be the domain of the lung in the Material (reference) configuration, and Ω = ***φ*** (Ω_0_, *t*) the deformed domain in the spatial (current) configuration that results from applying the deformation mapping $\varphi :\Omega_{0}\times R\to R^{3}$ . The deformation gradient tensor field $F:\Omega_{0}\times R\to R^{3\times 3}$ is defined as ***F*** ≔Grad ***φ*** (***X,t***), whose determinant *J* represents the volumetric change between Material and spatial configurations of an infinitesimal domain, i.e.,

$$J≔\frac{d\Omega }{d\Omega_{0}}=\text{det}F.$$ (1)

Let ϕ:Ω×R→R be the spatial porosity, defined as the fraction of gas volume over a reference volume in the current configuration. Then, the alveolar air and tissue differential volumes at a point ***x*** in the current configuration are given by *ϕ*(***x***, *t*)*d*Ω and (1 − *ϕ*(***x***, *t*))*d*Ω respectively. We also consider the Material porosity field $\Phi :\Omega_{0}\to R$ , which can be computed *via* a pull-back operation as Φ≔*J*(*ϕ*◦*φ*).

Within the composite porous medium that represents the lung functional domain, also termed lung parenchyma, alveolar gas and alveolar tissue coexist, and are mechanically coupled by means of conservation laws (MacMinn et al., 2016). We note that, in simplifying the analysis, other components found in the lung parenchyma such as blood and blood vessels, among other, are assumed to be part of the tissue phase (Berger et al., 2016). In the absence of sources or sink terms, the local form of mass conservation for alveolar air and tissue compartments can be expressed by

$$\frac{\partial \left(\rho_{a}\phi \right)}{\partial t}+\text{div}\left(\rho_{a}\phi v_{a}\right)=0\;\text{in }\Omega \times R,$$ (2)

$$\frac{\partial \left(\rho_{t}\left(1-\phi \right)\right)}{\partial t}+\text{div}\left(\rho_{t}\left(1-\phi \right)v_{t}\right)=0\;\text{in }\Omega \times R,$$ (3)

where $\rho_{a}:\Omega \times R\to R$ and $\rho_{t}:\Omega \times R\to R$ are the alveolar air and tissue spatial density fields, respectively. Further, $v_{a}:\Omega \times R\to R^{3}$ , $v_{t}:\Omega \times R\to R^{3}$ are the alveolar air and tissue spatial velocity fields, respectively. Based on these definitions, the spatial airflow field $q:\Omega \times R\to R^{3}$ can be obtained as

$$q≔\phi \left(v_{a}-v_{t}\right).$$ (4)

The linear momentum balance for the porous composite (parenchyma) takes the form (Coussy, 2004; Vuong et al., 2015)

$$\text{div}\sigma +\rho_{t}\left(1-\phi \right)\left(b-a_{t}\right)+\rho_{a}\phi \left(b-a_{a}\right)=0\;\text{in }\Omega \times R$$ (5)

where $\sigma :\Omega \times R\to R^{3\times 3}$ is the Cauchy stress tensor for the composite medium and $b:\Omega \times R\to R^{3}$ is the spatial body (gravity) force density. Further, $a_{a}:\Omega \times R\to R^{3}$ and $a_{t}:\Omega \times R\to R^{3}$ are the spatial acceleration fields for the alveolar air and tissue phases, respectively.

Following Berger et al. (2016), we neglect inertial terms and viscous stresses, and assume the incompressibility of both gas and tissue phases. These assumptions imply that both *ρ*_*a*_ and *ρ*_*t*_ are constant fields. We note that these conditions do not prevent the tissue phase to deform, as gas can enter or exit the composite domain during deformation, rearranging the pore structure and changing the local porosity field (MacMinn et al., 2016). Under these assumptions, Eqs 2, 3, 5 can be rewritten as

$$\frac{\partial \phi }{\partial t}+\text{div}\left(\phi v_{a}\right)\;\text{in }\Omega \times R,$$ (6)

$$\frac{\partial \left(1-\phi \right)}{\partial t}+\text{div}\left(\left(1-\phi \right)v_{t}\right)\;\text{in }\Omega \times R,$$ (7)

divσ+ρb=0in Ω×R, (8)

with *ρ* = (1 − *ϕ*)*ρ*_*t*_ + *ϕρ*_*a*_ the spatial composite density.

We will be interested in expressing the balance laws defined above in terms of Material fields defined on the Reference configuration Ω_0_. To this end, we consider the Material alveolar airflow field $Q:\Omega_{0}\times R\to R^{3}$ , which can be determined from a pull-back of its spatial counterpart defined in Eq. 4, which yields

$$Q=J\left(F^{-1}q\right)○\varphi .$$ (9)

Further, we consider the first Piola-Kirchhoff stress tensor field $P:\Omega_{0}\times R\to R^{3\times 3}$ , whose relation to the Cauchy stress tensor field is given by the Piola transformation, which reads

$$\sigma =J^{-1}PF^{T}.$$ (10)

Using these definitions, and *via* pull-back of the spatial balance expressions, it can be shown that the Material form of the gas mass conservation reads (Coussy, 2004; MacMinn et al., 2016)

$$\frac{\partial \Phi }{\partial t}+\text{Div}Q=0\;\text{in }\Omega_{0}\times R,$$ (11)

and the Material linear momentum balance for the composite reads

$$\text{Div}P+RB=0\;\text{in }\Omega_{0}\times R,$$ (12)

where *R*≔*J*(*ρ*◦ ***φ***) is the Material composite density and ***B*** = ***b*** ◦ ***φ*** is the Material body force density field.

### 2.2 Constitutive models for alveolar tissue and airflow

Based on standard arguments of the mixture theory of porous media, we consider the composite (parenchyma) Cauchy stress tensor to be

$$\sigma =\sigma^{\prime }-p_{\mathrm{alv}}I,$$ (13)

where $\sigma^{\prime }:\Omega \to R^{3\times 3}$ is the effective (tissue) Cauchy stress tensor, $p_{\mathrm{alv}}:\Omega \times R\to R$ is the alveolar air (pore) pressure field, and ***I*** the identity tensor. Using Eq. 10, the Material form of the stress decomposition yields (Coussy, 2004; Li et al., 2004; Sun et al., 2013)

$$P=P^{\prime }-JP_{\mathrm{alv}}F^{-T},$$ (14)

where $P^{\prime }:\Omega_{0}\times R\to R^{3\times 3}$ is the effective first Piola-Kirchoff stress tensor field and *P*_*alv*_≔*p*_*alv*_◦*φ*. In addition, for a solid phase made of a hyperelastic material we have (Chapelle and Moireau, 2014)

$$P^{\prime }=\frac{\partial W}{\partial F},$$ (15)

where $W:R^{3\times 3}\to R$ the strain energy function that characterizes the mechanical behavior of the alveolar tissue in the parenchyma. In this study, we consider five constitutive models specifically developed for describing the parenchyma mechanical response of human lungs (Ma et al., 2013; Berger et al., 2016; Yoshihara et al., 2017) and Wistar rats (Rausch et al., 2011; Birzle et al., 2019). These tissue models were chosen because they represent the state of the art in lung tissue modeling. The expressions for the strain-energy density and parameter values that approximate experimental observations in lung tissue are included in Table 1. Given the right Cauchy-Green tensor

$$C≔F^{T}F,$$ (16)

we consider the deformation invariants

$$I_{1}≔\text{tr}C,$$ (17)

$$I_{2}≔\frac{1}{2}\left[{\left(\text{tr}C\right)}^{2}-\text{tr}C^{2}\right],$$ (18)

$$I_{3}≔\text{det}C,$$ (19)

And we note that *I*_3_ ≡ *J* ^2^. Since all constitutive models considered in this work assume an isotropic solid, their strain energy densities can be written in terms of these three invariants, as reported in Table 1.

**TABLE 1:** Constitutive models for lung parenchyma: expressions for strain-energy densities and material parameters.

CM	Study	Strain energy functions	Parameters
CM1	Berger et al. (2016)	$W\left(C\right)=\frac{\mu }{2}\left(I_{1}-3\right)+\frac{\lambda }{4}\left(J^{2}-1\right)-\left(\mu +\frac{\lambda }{2}\right)\text{ln}\left(J-1+\phi_{0}\right)$	*μ* = 0.2808 kPa
			*λ* = 0.4212 kPa
			*ϕ*_0_ = 0.99
CM2	Ma et al. (2013)	$W\left(C\right)=c\;\text{exp}\left(a{\left(\frac{I_{1}}{2}-\frac{3}{2}\right)}^{2}+b\left(\frac{I_{2}}{4}-\frac{I_{1}}{2}+\frac{3}{4}\right)\right)$	*a* = 0.43
			*b* = −0.6
			*c* = 2 kPa
CM3	Birzle et al. (2019)	$W\left(C\right)=c\left(I_{1}-3\right)+\frac{c}{\beta }\left(I_{3}^{-\beta }-1\right)+c_{1}{\left(I_{1}I_{3}^{-1/3}-3\right)}^{d_{1}}$ $\;\;+c_{3}{\left(I_{3}^{1/3}-1\right)}^{d_{3}}$	*c* = 0.3567 kPa
			*β* = 1.075
			*c*_1_ = 0.2782 kPa
			*c*_3_ = 5.766 ⋅ 10^–3^ kPa
			*d*_1_ = 3
			*d*_3_ = 6
CM4	Yoshihara et al. (2017)	$W\left(C\right)=c\left(I_{1}-3\right)+\frac{c}{\beta }\left(I_{3}^{-\beta }-1\right)$	*c* = 1.298 kPa
			*β* = 0.75
CM5	Rausch et al. (2011)	$W\left(C\right)=c_{\text{quad}}{\left(I_{1}I_{3}^{-1/3}-3\right)}^{2}+c_{\text{cub}}{\left(I_{1}I_{3}^{-1/3}-3\right)}^{3}$	*c*_quad_ = 4.1 kPa
		$\;\;+\frac{\kappa }{4}\left(-2\text{ln}J+J^{2}-1\right)$	*c*_cub_ = 20.7 kPa
			*κ* = 16.5 kPa

Alveolar airflow inside the porous parenchyma is assumed to follow Darcy’s law, which in the case of a Newtonian fluid takes the form (Choo, 2018)

$$q=\frac{k}{\eta }\left[-\text{grad}\left(p_{\mathrm{alv}}\right)+\rho_{a}b\right],$$ (20)

or equivalently, in Material form, reads

$$Q=\frac{1}{\eta }JF^{-1}\kappa F^{-T}\left[-\text{Grad}\left(P_{\mathrm{alv}}\right)+\rho_{a}F^{T}B\right],$$ (21)

where $\kappa :\Omega_{0}\to R^{3\times 3}$ is the intrinsic permeability tensor and *η* is the dynamic viscosity of the gas. For a medium with isotropic permeability we consider ***κ*** = *κ* ***I*** and define permeability or conductivity as *k*≔*κ*/*η*. Following Berger et al. (2016), in our simulations we considered *k* = 10^4^ mm^2^/kPa⋅s.

### 2.3 Strong and weak formulations, and computational model construction

Let Γ_0_ be the boundary of the reference configuration Ω_0_. We assume that Γ_0_ admits the partition (Li et al., 2004; Sun et al., 2013; Vuong et al., 2015)

$$\Gamma_{\varphi }\cup \Gamma_{T}=\Gamma_{0}\;\;\;\;\Gamma_{\varphi }\cap \Gamma_{T}=\emptyset ,$$ (22)

$$\Gamma_{P}\cup \Gamma_{Q}=\Gamma_{0}\;\;\;\;\Gamma_{P}\cap \Gamma_{Q}=\emptyset ,$$ (23)

where Γ_*φ*_, Γ_*T*_, Γ_*P*_ and Γ_*Q*_ are the boundaries of prescribed deformation mapping, tractions, alveolar pressures, and alveolar airflow, respectively. Considering these boundary conditions, initial conditions on the unknown fields, and governing Eqs 11, 12, the strong Material poroelastic formulation of the lung mechanics problem can be stated as

Find $\varphi \in C^{2}\left(\Omega_{0}\times \left[0,T\right],R^{N}\right)$ and $P_{\mathrm{alv}}\in C^{2}\left(\Omega_{0}\times \left[0,T\right],R\right)$ such as:

$$\text{Div}\left(P\right)+RB=0\;\text{in }\Omega_{0}\times \left(0,T\right]$$ (24)

$$\frac{\partial \Phi }{\partial t}+\text{Div}\left(Q\right)=0\;\text{in }\Omega_{0}\times \left(0,T\right]$$ (25)

$$\varphi =\varphi_{0}\;\text{in }\Omega_{0}$$ (26)

$$P_{\mathrm{alv}}=P_{0}\text{in }\Omega_{0}$$ (27)

$$\varphi =\bar{\varphi }\;\;\text{on }\Gamma_{\varphi }\times \left(0,T\right]$$ (28)

$$P\cdot N=\bar{T}\;\text{on }\Gamma_{T}\times \left(0,T\right]$$ (29)

$$P_{\mathrm{alv}}=\bar{P}\;\text{on }\Gamma_{P}\times \left(0,T\right]$$ (30)

$$Q\cdot N=\bar{Q}\;\text{on }\Gamma_{Q}\times \left(0,T\right].$$ (31)

where ***P*** y ***Q*** are given by constitutive Eqs 14, 21, $\varphi_{0}:\Omega_{0}\to R^{3}$ and $P_{0}:\Omega_{0}\to R$ are the initial-value fields for the deformation mapping and alveolar pressure, and $\bar{\varphi }$ , $\bar{T}$ , $\bar{P}$ , $\bar{Q}$ are the prescribed deformation mapping, prescribed traction, prescribed alveolar pressure, and prescribed alveolar airflow fields, respectively.

Considering that water content in parenchymal tissue, which includes intracellular, interstitial, and blood water, can represent up to 80% of its mass (Lange and Schuster, 1999), we assume the tissue phase to be incompressible (Kowalczyk, 1993; Berger et al., 2016; Yoshihara et al., 2017). Further, we note that tissue incompressibility implies that (Coussy, 2004)

$$\Phi =J+\Phi_{0}-1,$$ (32)

where Φ_0_ is the initial Material porosity field.

To construct the weak formulation of the lung mechanics problem, we define the spaces of trial functions $S_{\varphi }$ , $S_{P}$ for the primary unknown fields ***φ*** and *P*_*alv*_ respectively; and their corresponding test function spaces $V_{\varphi }$ , $V_{P}$ as

$$S_{\varphi }≔\left\{\varphi :\varphi \in H^{1}\left(\Omega_{0},R^{3}\right);\;\varphi =\bar{\varphi }\;\;\text{on}\;\Gamma_{\varphi }\right\}$$ (33)

$$V_{\varphi }≔\left\{\eta :\eta \in H^{1}\left(\Omega_{0},R^{3}\right);\;\eta =0\;\;\text{on}\;\Gamma_{\varphi }\right\}$$ (34)

$$S_{P}≔\left\{P_{\mathrm{alv}}\in H^{1}\left(\Omega_{0},R\right);\;P_{\mathrm{alv}}=\bar{P}\;\text{on}\;\Gamma_{P}\right\}$$ (35)

$$V_{P}≔\left\{q\in H^{1}\left(\Omega_{0},R\right);\;q=0\;\text{on}\;\Gamma_{P}\right\}.$$ (36)

Following a standard Galerkin approach, we multiply governing Eqs 24, 25 by test functions $\eta \in V_{\varphi }$ and $q\in V_{P}$ respectively, apply integration by parts theorem where appropriate, and use boundary conditions given by Eqs 29 and 31 to obtain the weak formulation, which reads

Find $\left(\varphi ,P_{\mathrm{alv}}\right)\in S_{\varphi }\times S_{P}$ such that $\forall \;\left(\eta ,q\right)\in V_{\varphi }\times V_{P}$ :

$$\int_{\Omega_{0}}P:\text{Grad}\;\eta \;d\Omega_{0}-\int_{\Omega_{0}}RB\cdot \eta \;d\Omega_{0}-\int_{\Gamma_{T}}\bar{T}\cdot \eta \;d\Gamma_{T}=0$$ (37)

$$\int_{\Omega_{0}}\frac{\partial \Phi }{\partial t}\;q\;d\Omega_{0}-\int_{\Omega_{0}}\;Q\cdot \text{Grad}\;q\;d\Omega_{0}+\int_{\Gamma_{Q}}\;\bar{Q}\;q\;d\Gamma_{Q}=0.$$ (38)

Spatio-temporal discretization of the weak formulation was carried out using a backward-Euler time integration scheme and a standard Galerkin finite-element (FE) discretization, which yields a dynamic multi-field FE formulation (Hurtado et al., 2017a; Hurtado and Zavala, 2021). The computational implementation was performed using the FEniCS library (Alnaes et al., 2012), considering a Taylor-Hood (P2-P1) element technology.

### 2.4 Lung domain discretization and implementation of boundary conditions

**FIGURE 2:**
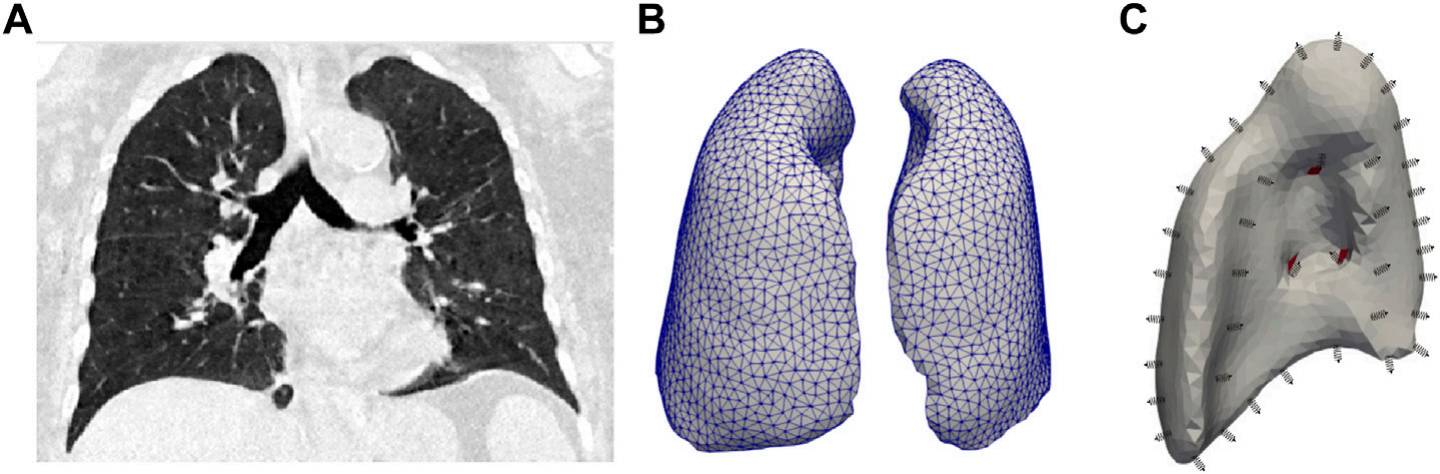
**(A)** CT image of the lung considered as the reference configuration. **(B)** Mesh for the finite element model. **(C)** Boundaries considered for right lung. The airways boundary is shown in red.

The domain of the whole-lung computational model was determined from a 3D computed-tomography (CT) image of the thorax of a normal human subject previously reported in the literature (Hurtado et al., 2017b), see Figure 2A. The CT image was acquired during the end of expiration (resting condition), which we assumed to be the Reference configuration of the lung. To create the FE lung mesh, we processed the image as described in previous contributions (Hurtado et al., 2016). In brief, the lung domain was identified from the original CT image dataset using the image segmentation tools included in the ITK Snap library (version 3.6.0) (Yushkevich et al., 2006). Based on this segmented mask image, a tetrahedral mesh was generated using the Computational Geometry Algorithms Library (Fabri and Pion, 2009), see Figure 2B for a graphical representation of the resulting lung mesh.The boundary of the lung domain was partitioned into the airways surface and the visceral pleura surface that lines the remaining lung surface. The airways boundary was determined by considering the surface encompassing bifurcations from the mediastinal surface down to the lobar bronchi. Smaller airways in subsequent branches were considered to be part of the lung parenchyma domain. The visceral pleura surface was defined as the complement of the airways surface. The following boundary conditions were considered:

1) Inflation and deflation: The air is supplied or expelled by a prescribed pressure condition $\bar{P}$ or prescribed airflow $\bar{Q}$ at the airways cross-section boundary, see Figure 1. The nature of this boundary condition depended on the application under consideration. In the case of pressure-controlled mechanical ventilation, pressure was prescribed on this boundary. Volume-controlled mechanical ventilation considers a prescribed airflow during inspiration and a prescribed pressure during the expiratory phase. For the simulations of the supersyringe method, airflow is prescribed on this boundary. From now on, we will refer to the airway boundary as Γ_aw_, regardless of the prescribed boundary condition.
2) Chest-wall effect: To model the mechanical interaction of the lung with the chest wall and mediastinum as well as the interaction between the airway wall and lung tissue, we considered spring elements acting in the direction normal to the surface over the visceral pleural surface and on the airway surface, see Figure 2C. The boundary traction was determined following a Robin boundary condition of the form

$$\bar{T}\left(X\right)=K_{\text{s}}\left\{\varphi \left(X\right)-X\right\},$$ (39)

with *K*_s_ a stiffness density constant, whose value is chosen to be *K*_s_ = 80 ⋅ 10^–3^ kPa/mm, which delivers a physiological response of the lung. We chose this value to represent an approximate chest-wall compliance value of 200 ml/cm H_2_O for any constitutive model used, which corresponds to values observed in normal subjects (Lumb, 2017). A discussion of this choice is presented later (see Figure 9 and the corresponding discussion).

In addition, we note that since this study focuses on analyzing the interaction between the lung and the ventilator using suitable boundary conditions, the body force field ***B*** is set equal to zero, i.e., the influence of gravity is neglected in our simulations.

### 2.5 Modeling the lung response to pressure-controlled ventilation

Invasive mechanical ventilation is a lifesaving treatment for acute respiratory distress syndrome patients. During mechanical ventilation, the lungs of a patient are connected *via* an endotracheal tube to a ventilator device that performs the respiratory function (Walter et al., 2018). The ventilator controls the air pressure applied to the airways or the amount of air that is pumped in every breath cycle. In this work, we consider two standard modes of invasive mechanical ventilation: pressure-controlled ventilation (PCV) and volume-controlled ventilacion (VCV).

The PCV mode is a pressure-targeted, time-cycled mode of operation, where the ventilator provides all the work of breathing, and the airflow and volume are dynamically adjusted to achieve the target airway pressure levels (Ashworth et al., 2018). During the inspiratory phase of PCV, air flows into the lung until a peak inspiratory pressure (PIP) value is reached, and then a flat pressure profile (square wave) is maintained for the rest of the inspiratory time (McKibben and Ravenscraft, 1996; Nichols and Haranath, 2007). After this, during the expiratory phase, the pressure is rapidly reduced to a set level of positive end-expiration pressure (PEEP) (Singer and Corbridge, 2011), which without loss of generality is assumed to be zero from here on.

To resemble this setup, in our finite-element simulations, we prescribe a pressure $\bar{P}$ on the airways boundary (see Figure 2C), which mimics the effect of the mechanical ventilator. At the beginning of inspiration, the prescribed airway pressure linearly increases until the PIP value is maintained during the inspiratory phase, i.e., $\bar{P}=\text{PIP}$ . Then, the airway pressure returns to zero during the expiratory phase $\left(\bar{P}=0\right)$ , after which another full respiratory cycle repeats. In our simulations, the PIP value was set as 6 cm H_2_0. The simulated ventilator protocol considers 1 s of inspiration (I) followed by 2 s of expiration (E) during each cycle, corresponding to a respiratory rate (RR) of 20 breaths per minute. These times were chosen because an inspiratory-expiratory ratio of I: E = 1 : 2 is recommended for normal lungs, as it resembles the respiratory cycle at rest (Bellani, 2022).

Once the finite-element lung simulations are carried out, the volume difference from the resting volume, i.e. the volume signal, is determined as

$${\text{V}}_{\text{sim}}\left(t\right)≔\int_{\Omega_{0}}J\;d\Omega_{0}-{\text{V}}_{\text{lung},0},$$ (40)

with

$${\text{V}}_{\text{lung},0}≔\int_{\Omega_{0}}\;d\Omega_{0}.$$ (41)

The simulated flow signal ${\dot{\text{V}}}_{\text{sim}}\left(t\right)$ is computed from integrating the alveolar airflow on the closing surface of the airways, i.e.

$${\dot{V}}_{\text{sim}}\left(t\right)≔\int_{\Gamma_{\text{aw}}}Q\cdot N\;d\Gamma_{\text{aw}},$$ (42)

which, in view of Eqs 25, 31, and 32, can be rewritten as

$${\dot{V}}_{\text{sim}}\left(t\right)=\frac{\partial V_{\text{sim}}}{\partial t}.$$ (43)

Further, we note that in our PCV simulations, the airway pressure signal is defined as

$${\text{P}}_{\text{aw, sim}}\left(t\right)≔\bar{P}.$$ (44)

### 2.6 Modeling the lung response to volume-controlled ventilation

In VCV mode, in each machine breath, a target amount of tidal volume (V_tidal_) is delivered using the same predetermined inspiratory flow–time profile, with the constant inspiratory flow being the most widely used breath delivery mode (Koh, 2007). In the conventional VCV, the expiratory valve is opened immediately after delivering the tidal volume. Modern ventilators allow an end-inspiratory pause to be included before expiration, where the ventilator sets the inspiratory flow to zero without opening the expiratory valve (Ball et al., 2015).

To simulate this ventilation mode, we consider that the V_tidal_ is supplied during the inspiratory phase by a constant prescribed airflow condition as

$$\bar{Q}=\frac{{\text{V}}_{\text{tidal}}}{{\text{A}}_{\text{aw}}\;{\text{T}}_{\text{ins}}},$$ (45)

where T_insp_ is the duration of inspiration and A_aw_ is the area of the airways boundary obtained as the surface integral over this boundary. Then, null airflow is imposed as $\bar{Q}=0$ during the end-inspiratory pause. Passive expiration was simulated by prescribing $\bar{P}=0$ on the airways boundary, similar to the PCV simulation. In our experiments, we considered a tidal volume of 500 ml, an inspiratory phase of 1 s, followed by a pause of 0.25 s, and an expiration of 2 s.

For the VCV mode, the ventilation signals can also be predicted by our model. During inspiration and pause, the volume is given simply as the integration of airflow over time

$${\text{V}}_{\text{sim}}\left(t\right)≔\int_{\left[0,t\right]}\bar{Q}\;d\tau ,$$ (46)

the flow signal is the prescribed airflow

$${\dot{V}}_{\text{sim}}\left(t\right)≔\bar{Q},$$ (47)

and the airway pressure signal is computed given as then average pressure on the airways boundary as

$${\text{P}}_{\text{aw, sim}}\left(t\right)≔\frac{1}{{\text{A}}_{\text{aw}}}\int_{\Gamma_{\text{aw}}}P_{\mathrm{alv}}\;d\Gamma_{\text{aw}}.$$ (48)

For the expiratory phase, in which airway pressure is prescribed, the simulated signals can be predicted by Eqs 40–44.

### 2.7 Estimation of respiratory-system compliance and resistance

In the clinical setup, and in particular for the management of patients under mechanical ventilation, the lung response is analyzed using the concepts of respiratory-system compliance and airway resistance (Hess, 2014). To this end, the response of the respiratory system is assumed to follow a single-compartment equation of motion, which reads (Bates, 2009)

$${\text{P}}_{\text{aw}}\left(t\right)=\frac{\text{V}\left(t\right)}{{\text{C}}_{\text{rs}}}+\text{R}\dot{\text{V}}\left(t\right),$$ (49)

where ${\text{P}}_{\text{aw}}:R\to R$ is the pressure signal at the airway opening, V:R→R is the volume signal, $\dot{\text{V}}:R\to R$ is flow signal, C_rs_ is the respiratory-system compliance and R is the airway resistance. According to the simulation conditions, in Eq. 49 a null positive end-expiratory pressure has been assumed, and the patient respiratory muscles are blocked and do not contribute to the respiratory effort.

The equation of motion can be used to estimate C_rs_ and R. To this end, for given ventilation signals V_sim_(*t*), ${\dot{\text{V}}}_{\text{sim}}\left(t\right)$ , we considerer the objective function (Bates, 2009)

$$S=\overset{\text{T}}{\underset{t_{i}=0}{\sum }}{\left[{\text{P}}_{\text{aw, sim}}\left(t_{i}\right)-\frac{{\text{V}}_{\text{sim}}\left(t_{i}\right)}{{\text{C}}_{\text{rs}}}+\text{R}{\dot{\text{V}}}_{\text{sim}}\left(t_{i}\right)\right]}^{2}.$$ (50)

Then, defining for convenience the respiratory-system elastance as E_rs_ = 1/C_rs_ and using a least squares fitting approach, the minimization problem to solve is

$$\underset{{\text{E}}_{\text{rs}}\in R^{+},\text{R}\in R^{+}}{\min}\;\overset{\text{T}}{\underset{t_{i}=0}{\sum }}{\left[{\text{P}}_{\text{aw, sim}}\left(t_{i}\right)-{\text{E}}_{\text{rs}}{\text{V}}_{\text{sim}}\left(t_{i}\right)+\text{R}{\dot{\text{V}}}_{\text{sim}}\left(t_{i}\right)\right]}^{2},$$ (51)

and can be shown that it is equivalent to solving the linear equation system

$$\left[\begin{array}{cc} \overset{\text{T}}{\underset{t_{i}=0}{\sum }}{\text{V}}_{\text{sim}}^{2}\left(t_{i}\right) & \overset{\text{T}}{\underset{t_{i}=0}{\sum }}{\text{V}}_{\text{sim}}\left(t_{i}\right){\dot{\text{V}}}_{\text{sim}}\left(t_{i}\right)\\ \overset{\text{T}}{\underset{t_{i}=0}{\sum }}{\text{V}}_{\text{sim}}\left(t_{i}\right){\dot{\text{V}}}_{\text{sim}}\left(t_{i}\right) & \overset{\text{T}}{\underset{t_{i}=0}{\sum }}{\dot{\text{V}}}_{\text{sim}}^{2}\left(t_{i}\right) \end{array}\right]\left[\begin{array}{c} {\text{E}}_{\text{rs}}\\ \text{R} \end{array}\right]=\left[\begin{array}{c} \overset{\text{T}}{\underset{t_{i}=0}{\sum }}{\text{V}}_{\text{sim}}\left(t_{i}\right)\;{\text{P}}_{\text{aw, sim}}\left(t_{i}\right)\\ \overset{\text{T}}{\underset{t_{i}=0}{\sum }}{\dot{\text{V}}}_{\text{sim}}\left(t_{i}\right)\;{\text{P}}_{\text{aw, sim}}\left(t_{i}\right) \end{array}\right],$$ (52)

from which the values C_rs_ = 1/E_rs_ and R describing the simulated respiratory system are obtained.

### 2.8 Modeling the supersyringe method: Construction of quasi-static pressure-volume lung curves

Quasi-static pressure-volume (P-V) curves are a gold-standard method to study lung mechanics in respiratory physiology research (West, 2012). The supersyringe method is used to construct these curves and consists of inflating the lung using controlled-volume incremental steps. In general, 50–100 ml of air are delivered into the lung in each step by moving a piston, with 2–3 s pauses between two successive inflations to reach quasi-static conditions. A similar procedure is used to construct deflation curves. Volume and pressure data are recorded, from which the P-V curves are then constructed (Harris, 2005; Stenqvist et al., 2008).

To simulate this standard technique in our lung model, we inflate the lung using a prescribed flow condition $\bar{Q}$ at the airway boundary, similar to the simulated inspiratory phase of VCV mode, see Eq. 45. The inflation curves are obtained by performing eight steps, where the inflation time (time in which the air enters through the movement of the piston) was 0.3 s, and the pause time was 2 s. During the inflation time, an airflow was imposed that would allow 100 ml to be supplied, while during the pause time, the airflow was zero. Once the inflation of the lung is finished, the deflation process is carried out considering similar times and flow magnitudes. In our P-V curves, the volume data was determined using Eq. 40, while pressure data was computed as the average pressure at the limit of the airway, see Eq. 48.

### 2.9 Estimation of chest-wall compliance

The compliance of the respiratory system, C_rs_, considers the contribution of the lung and the chest wall, whose relationship is given by (Hess, 2014)

$$\frac{1}{{\text{C}}_{\mathrm{rs}}}=\frac{1}{{\text{C}}_{\text{l}}}+\frac{1}{{\text{C}}_{\mathrm{cw}}},$$ (53)

where C_l_ is the lung compliance and C_cw_ is the chest wall compliance. In our simulations, the mechanical interaction between the chest wall and the lung was materialized by springs elements acting on lung surface. To assess the relation between the chest-wall compliance C_cw_ and the spring stiffness coefficient *K*_s_, we performed simulations that assumed a range of values, starting with the case of an unrestrained lung (*K*_s_ = 0). This last case allow us to determine the lung compliance C_l_. Then, from simulations using non-zero values of *K*_s_ we obtained the corresponding C_rs_, and from Eq. 53 we determined the corresponding C_cw_. These simulations were performed considering the PCV mode.

## 3 Results

**FIGURE 3:**
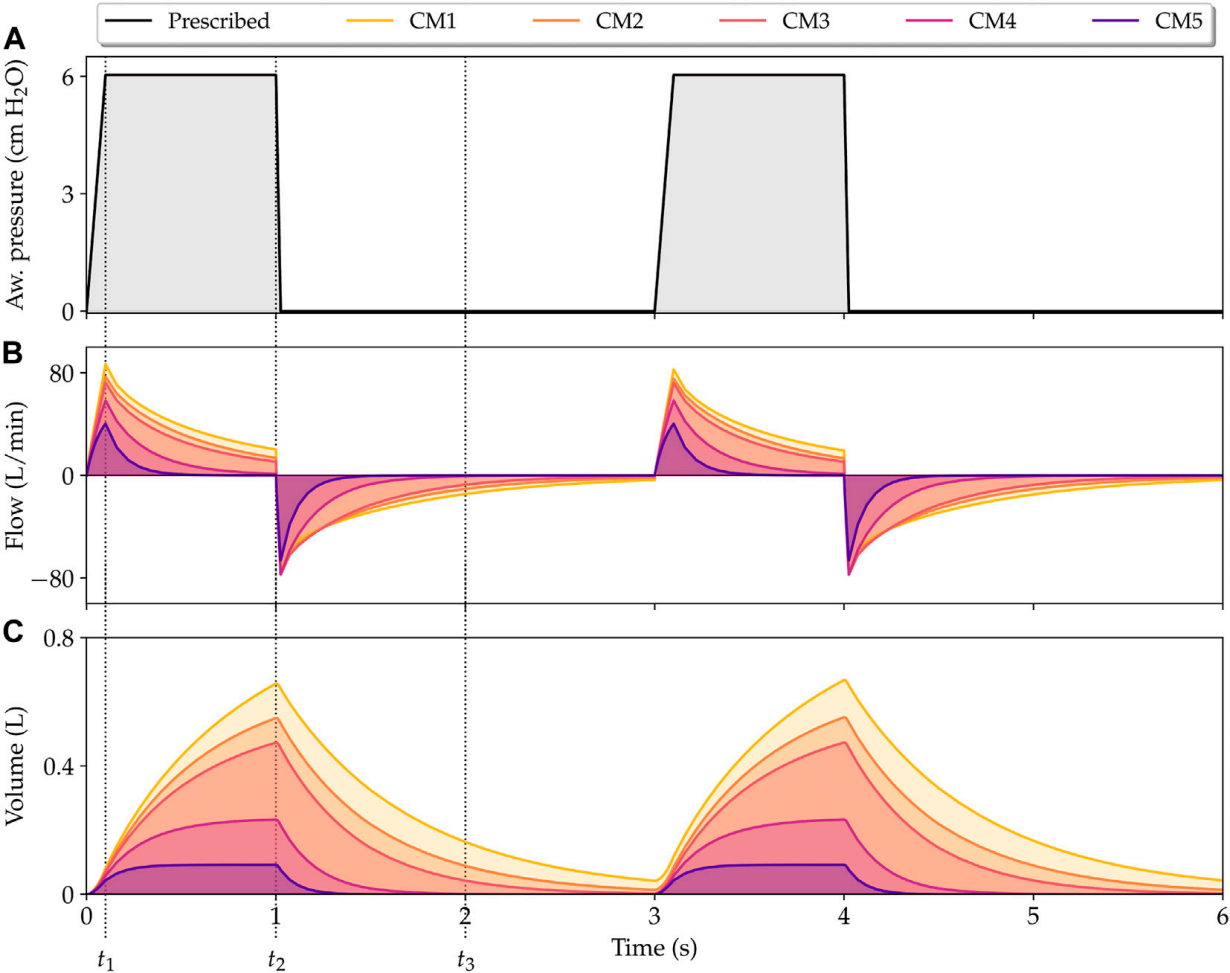
Simulation of lungs under pressure-controlled mechanical ventilation. Physiological signals that describe the time evolution of airway pressure **(A)**, flow **(B)**, and volume **(C)** are shown for all constitutive models considered in this work. *t*_1_, *t*_2_, and *t*_3_ represent the time instants for peak flow, peak volume, and half expiratory time, respectively.

Figure 3 shows the airway pressure, flow and volume signals predicted by the poroelastic model of the lung during PCV simulations. In this case, the airway pressure signal is prescribed and equal for all cases considered. The response of the lung model using the different constitutive laws described in Table 1 show significant variations in the volume and flow signals. In particular, the classical NeoHookean model (CM1) results in the highest peak flow and volume values. In contrast, the combined phenomenological model (CM5) displays the lowest peak lung flow and volume values. The cases of CM1 and CM5 form an envelope for all the other cases with different constitutive models. Further, we observe in the flow signals that different constitutive models can result in different exponential decays (time constants), with CM1 having the largest time constant, and CM5 the shortest.

Table 2 reports the respiratory system compliance and resistance for the five constitutive models studied obtained from fitting the equation of motion to the PCV finite-element simulations. The respiratory system compliance displays a strong dependence on the constitutive model choice, with the highest values being associated to the CM1 model. We also note that the CM5 model results in a compliance that is one order of magnitude smaller than that of CM1. In the case of the resistance, small variations are observed among the constitutive models studied.

**TABLE 2:** Respiratory system compliance and resistance for each model from the adjustment of the equation of motion to the computed waveforms.

CM	C_rs_ (ml/cm H_2_O)	R (cm H_2_O⋅L/s)
CM1	123	4.19
CM2	100	4.35
CM3	84	4.38
CM4	38	4.47
CM5	15	4.55

**FIGURE 4:**
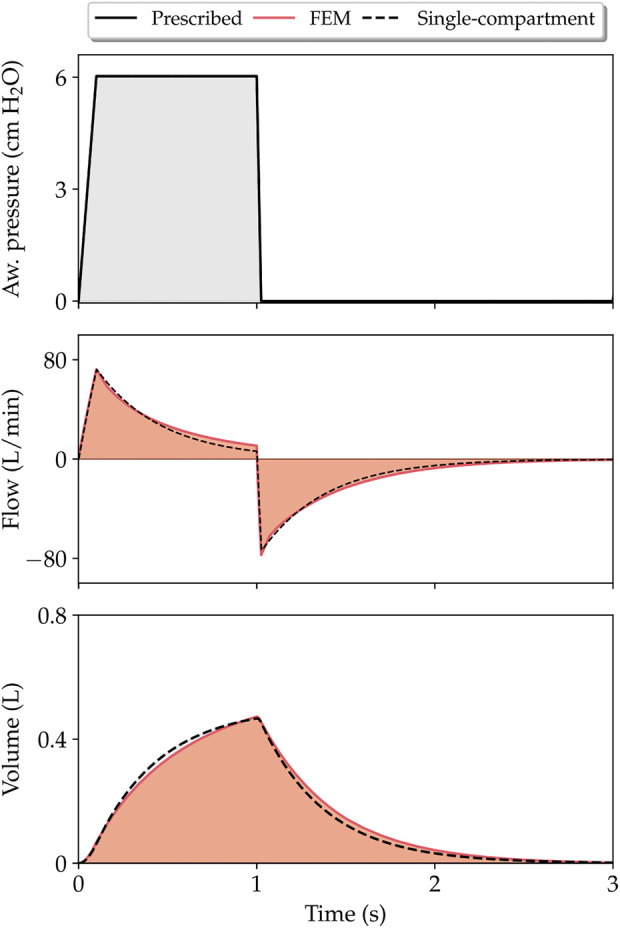
Comparison of respiratory variable evolution as predicted by finite-element simulations (CM3 model) and single-compartment model.

The predictions of lung respiratory signals generated by our finite-element model using the CM3 constitutive law were compared with those predicted by a single-compartment lung model described by Eq. 49 and C_rs_ and R given by Table 2, see Figure 4.

**FIGURE 5:**
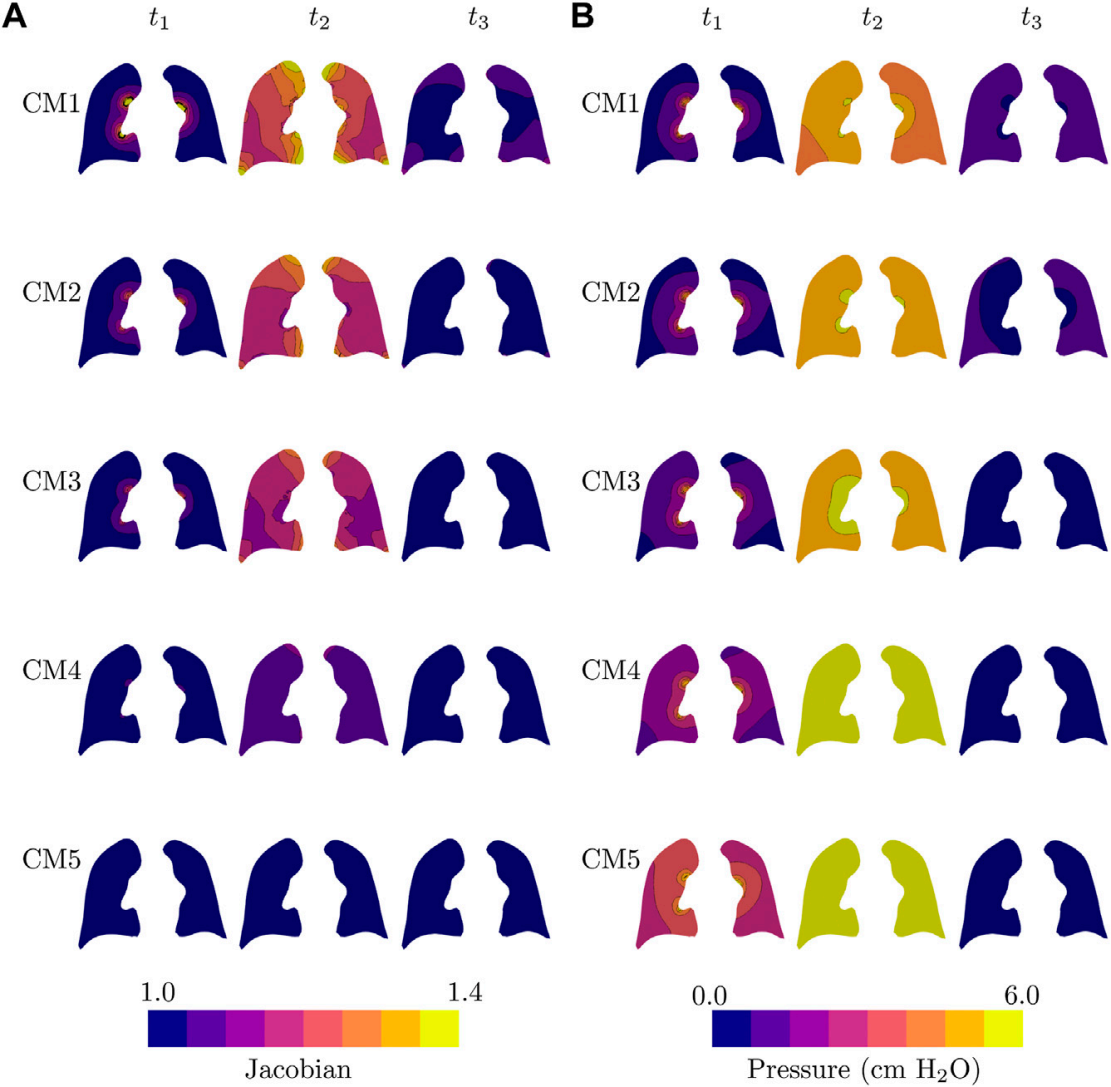
Temporal evolution of all lung models during one respiratory cycle of pressure-controlled mechanical ventilation: **(A)** jacobian field, and **(B)** alveolar pressure field. Fields are plotted in the current configuration.

The temporal evolution of the jacobian and alveolar pressure fields during PCV respiratory cycle for all the constitutive models considered is reported in Figure 5. We studied three key time instants to make comparisons between the models analyzed: time of peak flow (*t*_1_ in Figure 3), time of peak volume/end of inspiration (*t*_2_ in Figure 3), and time when half of the expiration subcycle has passed (*t*_3_ in Figure 3). For the same time instant, significant differences in the amplitude and distribution of these fields are observed. In the case of jacobian fields, which give an account of the local volumetric changes occurring in the lung, we note that during peak lung volume instant *t*_2_, the largest values are achieved for the CM1 model, and the smallest values for the case of CM5, see Figure 5A. In contrast, the highest levels of alveolar pressure are achieved by CM5 during the peak lung volume, whereas the lowest magnitudes at the same time instants are achieved by the CM1 simulation.

To study the evolution of stress fields in our lung simulations, we considered the effective hydrostatic and von Mises stress tensor invariants (Sarabia-Vallejos et al., 2019; Álvarez-Barrientos et al., 2021)

$$\sigma_{\text{hyd}}^{\prime }=\frac{1}{3}\text{tr}\sigma^{\prime },$$ (54)

and

$$\sigma_{\text{VM}}^{\prime }=\sqrt{\frac{3}{2}\sigma_{\text{dev}}^{\prime }:\sigma_{\text{dev}}^{\prime }},$$ (55)

with

$$\sigma_{\text{dev}}^{\prime }=\sigma^{\prime }-\sigma_{\text{hyd}}^{\prime }I.$$ (56)

**FIGURE 6:**
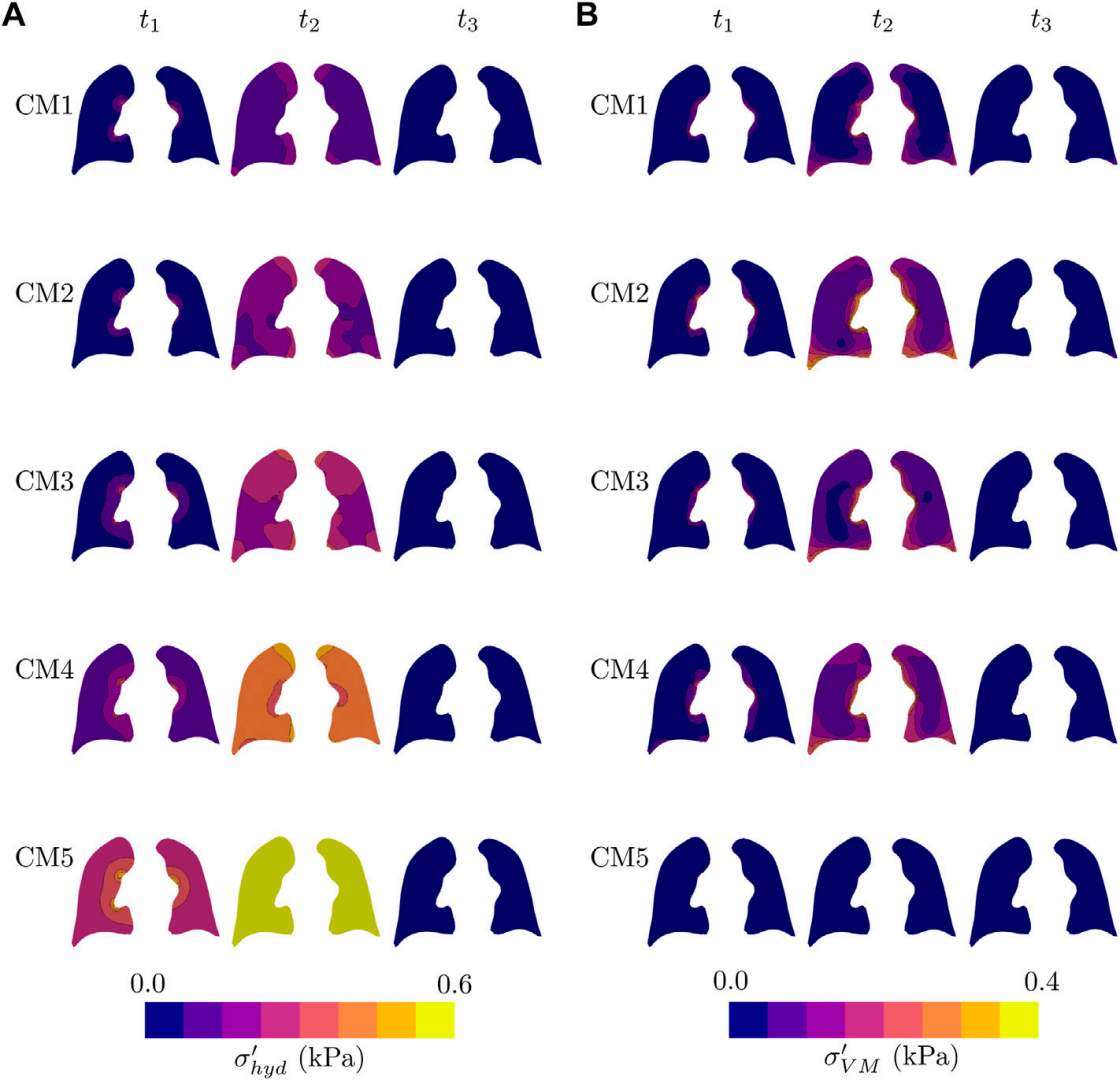
Temporal evolution of all lung models during one respiratory cycle of pressure-controlled mechanical ventilation: **(A)** Hydrostatic stress field, and **(B)** von Misses stress field. Fields are plotted in the current configuration.

Figure 6A reports the temporal evolution of the (effective) hydrostatic stress fields. Interestingly, at the time of peak volume (*t*_1_) we observe a significant variability in the levels of hydrostatic stress in the simulations considering different constitutive models, with CM5 resulting in the highest values. All the models present negligible hydrostatic stress in half of the expiration. Figure 6B shows the evolution of the (effective) von Mises stress. Simulations using CM1, CM2, CM3, and CM4 models predict heterogeneous distributions at peak lung volume, while CM5 does not display significant variations in the von Mises stress during the whole respiratory cycle.

**FIGURE 7:**
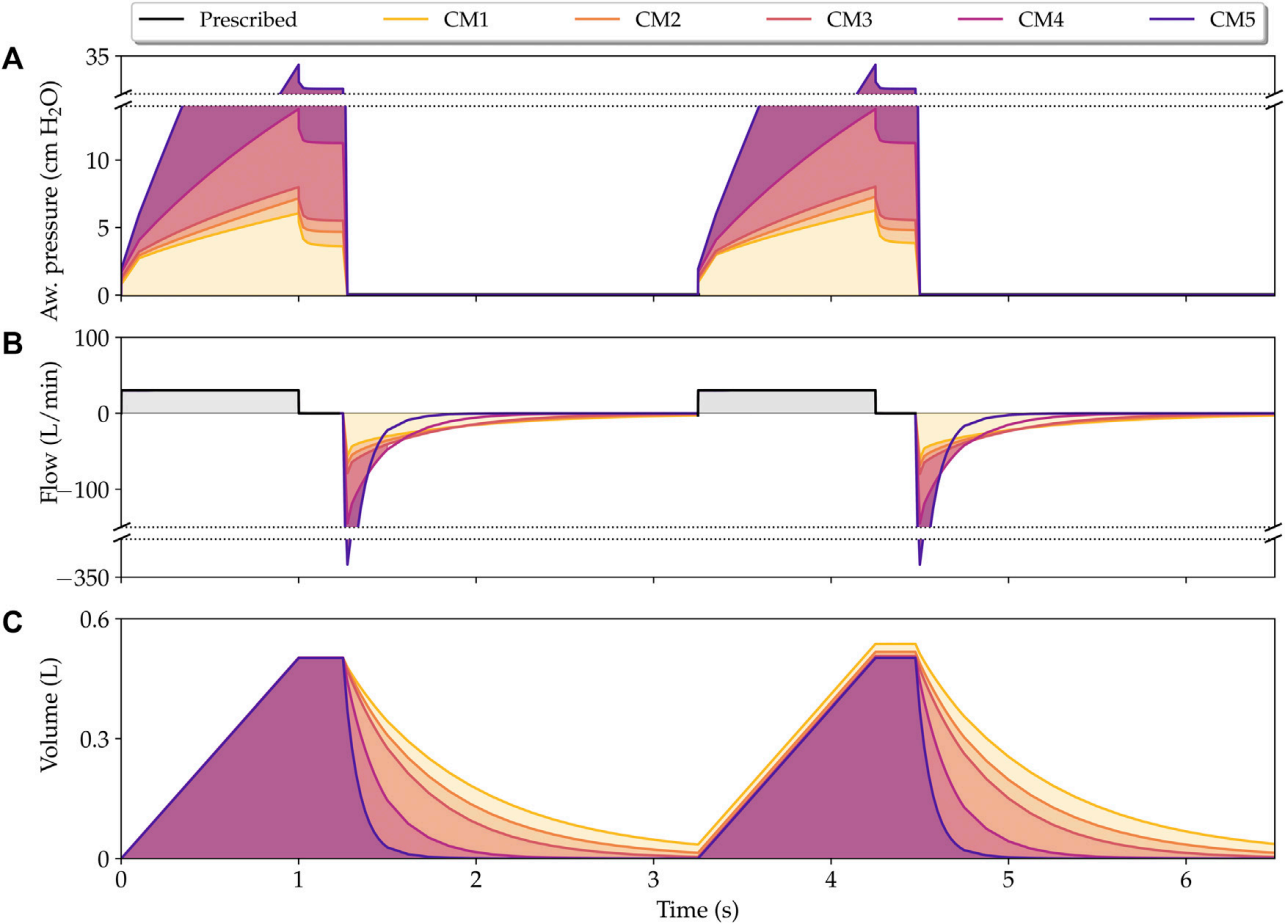
Simulation of lungs under volume-controlled mechanical ventilation. Physiological signals that describe the time evolution of airway pressure **(A)**, flow **(B)**, and volume **(C)** are shown for all constitutive models considered in this work. The *y*-axis was broken for the clarity of the plot.

The performance of our finite element model was also evaluated by predicting signals in the VCV mode. Figure 7 shows the airway pressure, flow, and volume signals for all the studied models. For this ventilation mode, the pressure during inspiration is not controlled by the mechanical ventilator, so its behavior is different for each constitutive model, with CM5 reaching the highest peak inspiratory pressure. During expiration, significant differences in flow and volume are observed between the constitutive models as a consequence of setting the pressure to zero.

**FIGURE 8:**
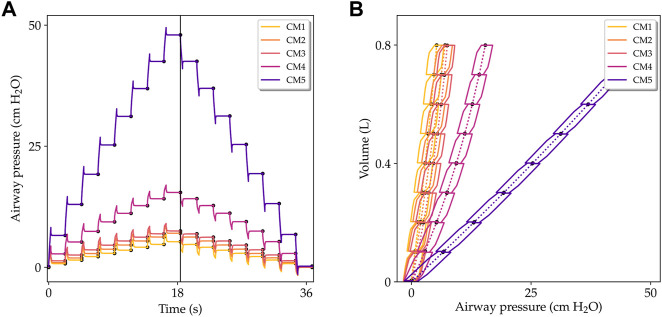
Simulation of lungs being studied with the supersyringe method. **(A)** Airway pressure *versus* time during the volume-controlled inflation-deflation protocol. **(B)** Pressure-volume curves for inflation and deflation processes. Solid lines show the pressure-volume evolution during the dynamic process. Dotted lines indicate the resulting quasi-static P-V curves for each constitutive model considered.

Figure 8 shows the results from simulations of the supersyringe method. The time evolution of the airway pressure as a response to increments of inspiratory volume is shown in Figure 8A. We note that the CM5 simulation results in airway pressure increments that are significantly higher than the increments observed for other constitutive models. The CM1 simulation displays the smallest pressure increments, and together with the CM5 case they form an envelope for all other constitutive models. Figure 8B shows the P-V lung curves for all models analyzed. The case of the CM1 simulation results in the highest static compliance (highest slope of the P-V curve), while the CM5 simulation results in the lowest static compliance.

**FIGURE 9:**
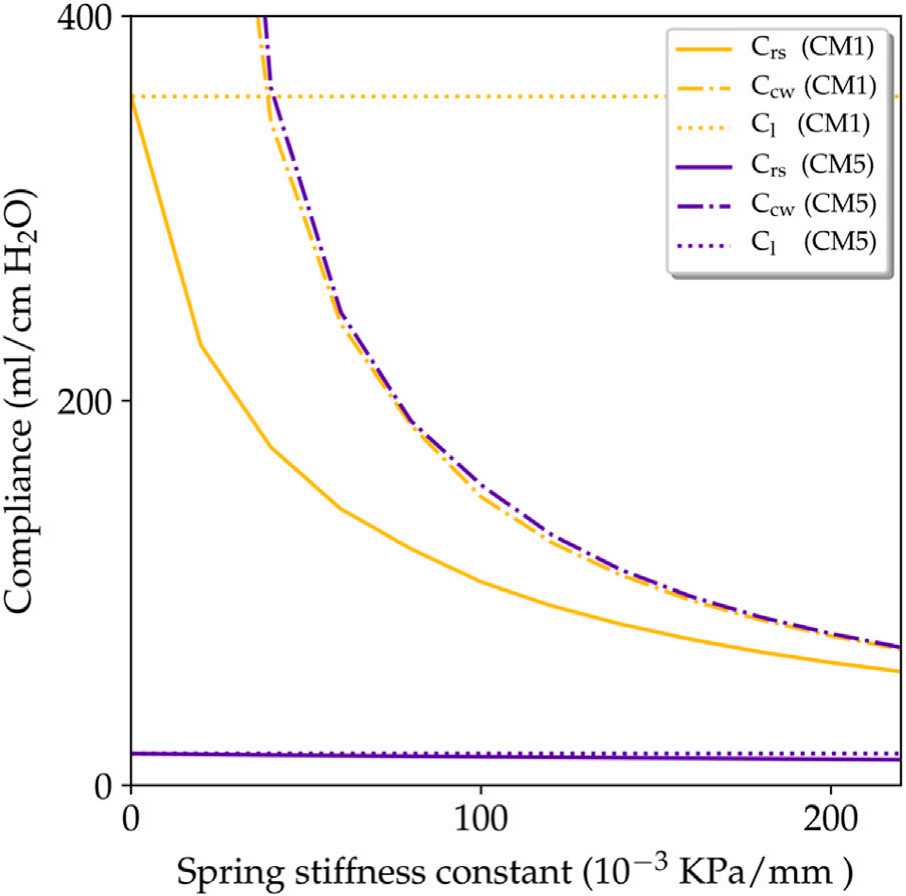
Influence of spring stiffness on the respiratory-system compliance C_rs_ and chest-wall compliance C_cw_.

The lung compliance C_l_ obtained using different constitutive models is reported in Table 3. The highest and lowest lung compliances are delivered by CM1 and CM5, respectively. These extreme compliance values differ by on order of magnitude. The influence of the spring stiffness density on the compliance of the respiratory system and of the chest wall is shown in Figure 9. For the CM1 simulation, we observe a strong inverse relation between C_rs_ and *K*_*s*_. In contrast, in the case of the CM5 simulation we observe a weak dependence between these two parameters, as the C_rs_ seems unaffected by changes in *K*_s_. For all constitutive models considered, the chest-wall compliance displays roughly the same inverse relation with respect to the spring stiffness constant.

**TABLE 3:** Lung compliance for each model (unrestrained lung).

CM	C_l_ (ml/cm H_2_O)
CM1	358
CM2	256
CM3	195
CM4	57
CM5	17

## 4 Discussion

In this work, we present a continuum poroelastic framework for the construction of high-fidelity subject-specific lung mechanics simulations. A distinctive advantage of our formulation is that, by construction, it seamlessly couples gas flow and local tissue deformation. We show that our computational lung model is suitable for simulations of the interaction between a mechanical ventilator on a PCV mode and the lungs of a patient, for which we recover key physiological signals such as the flow and volume, see Figure 3. Remarkably, as we discuss below, the simulation signals predict the waveform and many of the distinctive features typically observed in signals from real patients connected to a ventilator, see (Hess, 2014; Major et al., 2018) for a complete review on mechanical ventilation from a clinical perspective. During the inspiratory phase, the pressure imposed by the ventilator rapidly reaches a prescribed pressure level, which translates into a peak flow that is captured by our simulations, see time instant *t*_1_ in Figure 3. After reaching the prescribed pressure, the airway pressure is maintained to a constant level by the ventilator, which causes a gradual reduction and decelerated flow until the end of inspiration in patients (Rittayamai et al., 2015). This behavior is also predicted by our model, see time interval [*t*_1_, *t*_2_] in Figure 3. After the inspiratory phase, the ventilator abruptly releases the airway pressure during expiration, which quickly triggers a negative flow peak caused by the passive elastic recoil forces produced by the lung that eventually reaches a resting condition (zero flow) (Rittayamai et al., 2015). This expiratory process is also captured by our simulations, see the evolution after *t*_2_ in Figure 3. Interestingly, at the end of the expiratory phase, the CM1 model is not able to empty the lung volume, trapping small volumes of air, which can be observed in the volume signal at time instants 3 and 6 s. The opposite occurs with CM4 and CM5, which reach a zero flow before 2 s. CM2 and CM3 reach a null flow at the end of expiration, resulting in predictions that correspond well to the behavior of a normal lung under mechanical ventilation. These results suggest that CM1 may not be an adequate model to capture the lung elastic recoil mechanism, which is critical during the expiration phase.

To compare our simulations to clinical conditions, we determined key physiological parameters that are typically assessed in patients undergoing mechanical ventilation, such as the respiratory-system compliance and resistance, see Table 2. Regarding the prediction of respiratory-system compliance, we remark that all five constitutive models studied in this work deliver very different estimates, which may differ by an order of magnitude. Previous clinical studies indicate that acceptable values for C_rs_ range between 50 and 100 ml/cm H_2_O for MV patients (Hess, 2014). Taking these values as a reference, we conclude that for whole-lung simulations of a normal lung, the CM1 constitutive model delivers an abnormally soft mechanical response (too compliant). In contrast, the CM5 constitutive model results in an overly stiff behavior of the lung. Further, we conclude that lung tissue models CM2 and CM3 deliver respiratory-system compliance values that are in the reasonable range of normal compliance values. Regarding resistance, studies in normal human lungs connected to MV suggest that resistance is generally less than 10 cm H_2_O⋅L/s (Pham et al., 2017). The resistance values reported in Table 2 fall within this range. Interestingly, while the choice of constitutive model markedly affects the respiratory-system compliance, its impact on the resistance is low, as it can induce variations of less than 10%. This weak dependence is explained by the fact that in the continuum framework, alveolar flow is strongly driven by pressure gradients and modulated mainly by the permeability tensor, see Eq. 20. As an additional validation step, the lung flow and volume variables predicted by finite-element simulations were compared with traditional single-compartment models based on solving Eq. 49, see Figure 4. Interestingly, for a prescribed airway pressure signal, flow and volume waveforms and peak values predicted by these two models were very similar. This agreement confirms that the proposed poromechanical continuum framework captures the overall lung mechanical behavior that has long been represented by traditional single-compartment models, at the same time that it offers a connection between whole-organ response and regional mechanisms as we discuss next.

One of the salient features of our continuum framework is its ability to predict 3D fields of relevant physical quantities. The local volume change, represented by the jacobian field in Figure 5A, shows that CM1, CM2, and CM3 simulations predict for the end of inspiration (*t*_2_) a very non-uniform distribution of volumetric change, presenting the apical segments, and the areas near to the costophrenic and cardiophrenic angles the most significant deformations. In contrast, the CM5 simulation results in roughly no volumetric deformations during the ventilation cycle, as the Jacobian field at *t*_1_, *t*_2_ and *t*_3_ are homogeneous and close to 1.0 everywhere. The Jacobian of CM4 is also quite uniform, with small increases in the apical segment in *t*_2_. These observations are in line with the peak lung volumes observed in Figure 3. We remark that spatial heterogeneity in volumetric strain fields has been reported in human lungs using image-based strain analysis methods (Amelon et al., 2011; Hurtado et al., 2017b). However, a direct comparison may be inconsistent, as these studies were carried out in subjects breathing spontaneously and in maximal effort, which physiologically differs from lungs connected to MV. In the case of alveolar pressure, less heterogeneity is observed in the distribution predicted by the different constitutive models, see Figure 5B. For CM5, the alveolar pressure almost immediately after the ventilator pressure rise, reaching in *t*_1_ values close to half of the peak inspiratory pressure in much of the coronal view presented, while in *t*_2_ the maximum pressure is uniformly reached. A similar distribution in *t*_2_ is achieved by CM4, even though its values in *t*_1_ are lower than those in CM5. In contrast, CM1, CM2, and CM3 present a non-uniform alveolar pressure distribution in *t*_2_. From this, we note that the choice of the constitutive model also affects the pressure field distribution, although its effects seem to be less than the impact on the volume change.

The time evolution of stress fields was also studied in this work, see Figure 6. We observe that during peak lung flow (*t*_1_), the hydrostatic stress displays a radial gradient, with the area near the airways boundary presenting the highest values of hydrostatic stress, see Figure 6A. At the end of inspiration, a heterogeneous distribution is reached, with the zones with the most significant tension being the areas with bigger volumetric change (see Figure 5A). Interestingly, the stiffest model (CM5) achieves the highest hydrostatic stress, being highly uniform en *t*_2_. Also, in the midtime of the expiratory phase (*t*_3_), all the models present negligible hydrostatic stress in the coronal view reported. An analysis of the evolution of the von Mises stress shows a rise and fall in stress levels for all models during the respiratory cycle, with the notable exception of the highly rigid CM5, for which the deviatoric component of stress seems to be negligible and insensitive of the MV stimulus, see Figure 6B. The areas with the most significant values are those close to the lung surface; see the zones near to the edges in the coronal view at *t*_2_. From the above, we conclude that our model can capture not only the stresses related to volumetric changes in the lung but also the existence of shear-related stresses. However, for a healthy lung, these values appear to be less than the stresses associated with volumetric changes.

The interaction between the mechanical ventilator in VCV mode and the lungs of a patient was also analyzed by constructing the key physiological signals such as the airway pressure, flow, and volume, as shown in Figure 7. During the inspiratory phase, a fast rise in pressure is caused by the square wave of flow during the first few moments of inspiration, followed by a quasi-linear increase in airway pressure until it reaches the peak inspiratory pressure. Remarkably, the PIP reached by CM5 is extremely large compared to the other models, reaching more than 30 cm H_2_O, a value that, in previous studies (Vasilyev et al., 1995; Yang et al., 2011; Walter et al., 2018) has been associated with an upper limit of pressure for a protective mechanical ventilation. In this context, CM5 also does not seem to be a suitable model to model a healthy human lung in VCV as it requires very high pressures to be able to enter a (normal) tidal volume of 500 ml. Although the other models reach lower peak pressures, there are also considerable differences between them, which is justifiable given the difference in stiffness of each constitutive model, which can be represented in the form of compliance as shown in Table 2. After reaching PIP, thanks to flow restriction during the inspiratory pause, the lung comes to a quasi-static state, as observed in clinical mechanically ventilated patients (Ball et al., 2015). This quasi-static state is characterized by the decrease in peak inspiratory pressure to a steady state value known as plateau pressure (P_plat_). Interestingly, the drop from PIP to P_plat_ is similar for all constitutive models, despite the differences in stiffness and pressures achieved. According to clinical studies (Hess, 2014), this decrease is attributed to resistance. As previously noted, in this work, the resistance has mainly been taken into account through the permeability tensor, which has been chosen as a constant for all the constitutive models, explaining the same drop pressure. Also, we emphasize that in the VCV simulation, the system respiratory compliance and airway resistance can be estimated using the value of the pressure, flow, and volume variables during the inspiratory pause (see for example Singer and Corbridge (2009)) or employing the least squares fit approach presented. At the end of the inspiratory pause, the expiratory valve is open, and a negative peak flow is reached, which, consistent with the PIP value, is exceptionally high for CM5. Then, the volume reduces exponentially, with CM4 and CM5 being the fastest models to expel inspired air. In contrast, due to the elastic recoil discussed previously, CM1 traps air volumes at the expiration’s end, which results in an increase in volume during the second respiratory cycle. On the other hand, CM2 and CM3 seem to represent better the behavior of a normal human lung under the VCV conditions considered.

Our lung model was also studied in the simulation of traditional assessment techniques in respiratory physiology such as the supersyringe method for the construction of P-V curves (West, 2012). We remark that the simulation of airway pressure during the volume-controlled inflation and deflation phases shown in Figure 8 recovers many features observed in experimental setups (Harris, 2005; Ganzert et al., 2009). For example, during each inflation step, the airway pressure rapidly peaks, followed by an asymptotic decrease that reaches a steady-state airway pressure value, see Figure 8A. An opposite trend is observed during the deflation process. P-V curves resulting from this simulated experiment are collected in Figure 8B for all the constitutive models studied. The steady-state P-V curve, constructed by joining all the points that correspond to steady-state pressure, is shown in dotted line. We readily observe marked differences in static compliance, defined as the slope of steady-state P-V curves, that depends on the constitutive model employed. Similarly to the MV case, the CM1 simulation results in the most compliant (softest) case, whereas the CM5 model delivers the least compliant (stiffest) response. We further note that, in contrast to experimental P-V curves, the quasi-static curves of inflation and deflation are the same, and the simulated response does not capture the quasi-static hysteretic response of normal lungs (Escolar and Escolar, 2004; Steimle et al., 2011). This result is to be expected, as constitutive models considered in our poromechanical framework are hyperelastic, and no dissipative stress contributions have been included in the model. This represents an important limitation of the current contribution that should be addressed in future developments.

Figure 9 shows the relationship between the spring stiffness constant and the different compliance parameters, as predicted by our simulations. For the case of CM1, an inverse relation between *K*_s_ and C_rs_ was found, suggesting that the stiffness provided by the thoracic cage modulates the global elastic response of the respiratory system. In high contrast, the case of CM5 results in a respiratory-system compliance that is independent of the value of *K*_s_. This may be understood by examining Eq. 53, where a very low lung compliance, as that reported in Table 3, dominates over the chest-wall compliance. This in turn, forces C_rs_ respiratory-system to approach C_l_, which is indeed what we recover in Figure 9. Despite these large differences caused by the choice of constitutive model, we highlight that the chest-wall compliance C_w_ is not affected by the choice of constitutive model, see the dash-dotted lines in Figure 9. This independence allows to define the patient-specific chest-wall compliance only through the *K*_s_ parameter, which simplifies the personalization of lung models. Chest-wall compliance can be highly variable depending on the underlying pathology of each patient. For example, chest-wall compliance values of 200 ml/cm H_2_O have been reported for normal subjects (Lumb, 2017), while obese subjects have presented chest-wall compliance of 77 ml/cm H_2_O (Naimark and Cherniack, 1960). Our model can recover both cases using a value of *K*_s_ close to 80 ⋅ 10^–3^ kPa/mm and 200 ⋅ 10^–3^ kPa/mm, respectively.

This contribution represents a definite proof of concept that computational models of the lung can be used to simulate clinically-relevant procedures in respiratory medicine. Our work has several limitations that offer key opportunities for improvement and future developments. First, our framework models the interaction of the lung with surrounding structures by using distributed spring elements with the same stiffness. It is important to note that around the lung different muscles, organs, and bone structures will have different stiffness. In addition, our model does not consider the lubricating effect of the pleural fluid that lies in the pleural cavity between the lung and the chest wall. Recent works in the literature have modeled this interaction under quasi-static conditions by means of sliding contact elements (Patte et al., 2022b), which may constitute future additions to our lung model to account for the sliding mechanism. Second, in modeling the lung we have considered a stress-free reference configuration constructed from CT images of resting lungs. However, real lungs in resting conditions bear residual stresses that are caused by the transpulmonary pressure necessary to avoid alveolar collapse (Suki et al., 2011). Introducing residual stresses has been approached in former contributions (Tawhai et al., 2009; Berger et al., 2016; Patte et al., 2022b), but the validity of these approaches remains to be confirmed, as a definite experimental study of residual stresses in real lungs remains unexplored. Third, our simulations only consider hyperelastic constitutive models that neglect the hysteretic behavior of lung tissue due to alveolar surfactant (Andreassen et al., 2010). We note that surface-tension effects have been previously incorporate into micromechanical model of single alveolus (Denny and Schroter, 2000; Wiechert et al., 2009). Future developments may upscale these alveolar models into continuum formulations to account for surface-tension effects in the whole-lung response (Concha et al., 2018; Concha and Hurtado, 2020). Further, constitutive parameters were set constant throughout the lung, an assumption that may not adequate to capture the variability observed in clinical conditions. To further personalized the proposed model, parameter values and their spatial distribution could be estimated from available clinical data and image information using inverse-analysis techniques (Patte et al., 2022a). Fourth, while our model has been validated by comparing global parameters such as respiratory compliance and resistance to values reported in the literature, regional values of strain and stress predicted by our simulations remain to be validated. To the best of our knowledge, there are currently no studies that report regional deformation in normal lungs connected to mechanical ventilation. An interesting approach would be to simulate spontaneous breathing triggered by diaphragmatic motion and compare our model predictions to strain fields in normal human lungs determined from image registration (Amelon et al., 2011; Hurtado et al., 2017b). This poses the challenge of determining accurate boundary conditions for uncontrollable breathing efforts. Additional validation efforts may also consider the evolution of regional ventilation, which can be approximated by the porosity field. Finally, we note that our work has focused on clinical conditions where the respiratory activity is highly controllable. Future developments should study the applicability of our lung model to other respiratory conditions, e.g., spontaneous breathing. Given the uncontrolled nature of spontaneous breathing, extending our model to this case will required precise measurements of the dynamic boundary conditions acting on the lung surface, such as the pleural, diaphragmatic, and abdominal pressures. One promising approach to reaching this objective may be the use of organosynthetic lung simulators, which have been recently reported in the literature and enable precise measurements of these important physiological variables that are difficult to monitor *in vivo* (Horvath et al., 2020).

## Data Availability

The raw data supporting the conclusion of this article will be made available by the authors, without undue reservation.
